# Insight into the Antioxidant Effect of Fermented and Non-Fermented *Spirulina* Water and Ethanol Extracts at the Proteome Level Using a Yeast Cell Model

**DOI:** 10.3390/antiox10091366

**Published:** 2021-08-27

**Authors:** Jasmina Masten Rutar, Berta Cillero-Pastor, Ronny Mohren, Nataša Poklar Ulrih, Nives Ogrinc, Polona Jamnik

**Affiliations:** 1 Department of Environmental Sciences, Jožef Stefan Institute, Jamova 39, 1000 Ljubljana, Slovenia; jasmina.masten@gmail.com (J.M.R.); nives.ogrinc@ijs.si (N.O.); 2 Jožef Stefan International Postgraduate School, Jamova 39, 1000 Ljubljana, Slovenia; 3 The Maastricht MultiModal Molecular Imaging Institute (M4I), Maastricht University, Universiteitssingel 50, 6229 ER Maastricht, The Netherlands; b.cilleropastor@maastrichtuniversity.nl (B.C.-P.); r.mohren@maastrichtuniversity.nl (R.M.); 4 Biotechnical Faculty, University of Ljubljana, Jamnikarjeva 101, 1000 Ljubljana, Slovenia; natasa.poklar@bf.uni-lj.si

**Keywords:** *Spirulina*, lactic acid fermentation, *Saccharomyces cerevisiae*, ethanol, proteome, antioxidant, stress response

## Abstract

*Spirulina* is rich in various antioxidants and nutraceuticals and it has proven to be effective in the treatment of various pathological conditions. This study explores the antioxidant effect of fermented and non-fermented *Spirulina* extracts on the proteome level using the yeast *Saccharomyces cerevisiae* as a model organism. Yeast cells were treated with fermented *Spirulina* water extract (SV), non-fermented *Spirulina* water extract (NFV), fermented *Spirulina* ethanol extract (SE), and non-fermented *Spirulina* ethanol extract (NFE). Cell lysates were prepared, and label-free quantitative proteome analysis was performed. In SV, when compared to NFV samples, the levels of most differentially expressed proteins were upregulated. Alternatively, SE compared to NFE samples showed a significant downregulation for the majority of the analyzed proteins involved in different cellular processes. Additionally, a higher downregulation of stress response related proteins was observed in SE compared to NFE samples, while their abundance in SV samples increased compared to NFV. This study provided a global view, on a proteome level, of how cells cope with exogenous antioxidants and remodel their cellular processes to maintain metabolic and redox balance. Furthermore, it combined for the first time the analysis of different extract effect, including the contribution of lactic acid fermentation to the cell activity.

## 1. Introduction

*Spirulina* (*Arthrospira* spp.) is a *filamentous* cyanobacterium (blue-green algae) capable of photosynthesis. It is a very nutritious food containing various phytonutrients, antioxidants, nutraceuticals, and probiotics [1,2,3] and a good source of proteins (covering all essential and most non-essential amino acids) [4,5], vitamins, and minerals. It is especially rich in iron, gamma-linolenic fatty acid, B vitamins, and carotenoids. Due to its high content of nutrients, it has been proven experimentally to be effective in the treatment of many pathological conditions such as cardiovascular disease, hyperlipidemia, hepatotoxicity, and certain cancers, among others [6,7,8,9].

Fermentation of food products by lactic acid bacteria has been shown to improve the nutraceutical profile of foodstuffs. For example, lactic acid bacteria can degrade plant as well as cyanobacterial cell walls using enzymatic hydrolysis. This results in the production of molecules with high immunomodulatory, antioxidant, and anti-inflammatory biological activity by converting large organic compounds [10,11,12]. Lactic acid fermented *Spirulina* contains high amounts of polyphenols and phycocyanobilin and has higher protective activity, preventing cell damage by UVB radiation-induced oxidative stress. Furthermore, fermented *Spirulina* biomass has significantly higher antioxidant activity compared to non-fermented *Spirulina* biomass [13]. Studies have shown that after fermentation, the nutraceutical value of *Spirulina* evaluated through total phenolic and C-phycocyanin content, radical scavenging capacity, oxygen radical antioxidant capacity, protein fragmentation and ferric reducing antioxidant power, increases significantly, with the best results being achieved after 36 h of fermentation. After that time, the total antioxidant capacity diminishes, while the bioactive peptide, thermostable protein content and free methionine content increase, reaching a maximum at 72 h of active *Spirulina* biomass fermentation [10,14].

Different solvents (water, ethanol, methanol, DMSO, hexane, and petroleum ether) have been used to prepare *Spirulina* extracts for antioxidant, anticancer, antimicrobial, antiproliferative properties, protective effects against apoptotic cell death, and neuroprotective effects analysis due to differences in solubility of the various bioactive compounds [3,7,15,16,17,18,19,20,21]. For example, phycobiliproteins are soluble in water, whereas phenolic compounds and biologically active phytochemical substances such as sterols, tannins, flavonoids, reducing sugars, anthraquinone, chlorophyll-a, carotenoid pigments, and tocopherol have a higher solubility in alcohols. Ethanol and methanol extracts retain higher antiradical and antioxidant activity compared to water extracts, due to which it is possible to believe that alcohol-soluble components have the main antioxidant properties and are present in higher concentrations [15,16,22,23]. Conversely, *Spirulina* water extracts significantly reduce apoptotic cell death induced by free radicals [3].

Due to the feasible expression of proteins of any origin and easy RNA level manipulation, analysis of proteins and pathways is facilitated in yeast, whereas the same phenomena would be difficult to study in more complex organisms. Using yeast as a test organism also has many technical advantages over human cells. For instance, it has a fast life cycle and can grow in colonies on solid media or as dispersed cells in a liquid, and does not require expensive media [24]. Furthermore, apparent homologs can be identified in the human genome over the entire proteome, corresponding to 46% of the yeast proteome. Thus, yeast studies focused primarily on component parts can yield information that applies to the human counterparts [25], all of which makes yeast a model organism for researching basic human cellular processes, metabolic pathways, and cell stress response [26].

Our previous results have already shown the important role of lactic acid fermentation in enhancing the antioxidant effect of *Spirulina* when ethanol extracts were tested by measuring the intracellular oxidation level in the yeast *Saccharomyces cerevisiae*. Namely, a 19%- and 39%-decrease in intracellular oxidation level according to the control was observed when cells were treated with ethanol extracts of non-fermented and fermented *Spirulina*, respectively. In contrast, no changes in intracellular oxidation level were observed, when cells were exposed to water extracts of non-fermented as well as fermented *Spirulina* biomass. Additionally, total phenolic content (TPC) was determined, and it was higher in the water extracts of both non-fermented (17.31 mg equivalent of gallic acid/g DW) and fermented (11.67 mg eq. GA/g DW) *Spirulina* biomass compared to ethanol extracts. Thus, after fermentation a 33%-decrease of TPC was observed in water extracts, while ethanol extracts showed higher content (3.78 mg eq. GA/g DW) when compared to non-fermented *Spirulina* biomass extracts (2.60 mg eq. GA/g DW) [27]. In the present study, the difference between water and ethanol extracts and the role of lactic acid fermentation in the antioxidant activity of the extracts was further investigated at a proteome level using the same cell model organism. The yeast cells were in the stationary phase, which resemble cells of multicellular organisms in important aspects: (i) most of the energy comes from mitochondrial respiration, (ii) cells are in the G_0_ phase, (iii) oxidative damages accumulate over time [28] and have the same defense mechanisms as higher eukaryotes [29,30].

The current study provides an in-depth insight into the lactic acid fermentation of *Spirulina* and solvent choice for bioactive compounds extraction by providing results of *Spirulina* treatment effect on the proteome level of the yeast cells. To the best of our knowledge no studies have yet been published on the effect of *Spirulina* treatment on the yeast cell proteome level.

## 2. Materials and Methods

### 2.1. Lactic Acid Fermentation of Spirulina

*Lactobacillus plantarum* stock culture LMG 6907 (Institute of Dairy Science and Probiotics, Department of Animal Science, Biotechnical Faculty, Slovenia) in 20% glycerol (50 μL) was added to De Man, Rogosa, and Sharpe medium (20 mL) (MRS, Merck, Darmstadt, Germany) and incubated (150 rpm, 30 °C, overnight) on a rotary shaker until the late exponential phase was reached. The obtained broth was then centrifuged (14,000× *g*, 5 min) (2 mL) and washed twice with a physiological solution to obtain suspension for inoculation.

The freeze-dried *Spirulina* sample (2.47 g) was reconstituted to a total of 10 g by adding sterile ddH_2_O. The reconstituted sample was then mixed with the physiological solution (10 mL), inoculated with the *L. plantarum* suspension (200 μL, 1% (*v*/*v*) inoculum) and allowed to ferment (30 °C, 24 h). The fermented samples were then stored at −20 °C.

### 2.2. Spirulina Extract Preparation

In order to determine the effect that *Spirulina* biomass has on yeast cells before and after fermentation, both the fermented and non-fermented *Spirulina* broth were extracted with water and ethanol (96%). Two-stage extraction was performed to obtain higher yields. In the first stage, the *Spirulina* broth (8 g) was mixed with 12 mL of the extraction solvent (water or ethanol) and placed in a water bath (40 °C, 30 min) with constant shaking. The samples were then centrifuged (6000 rpm, 10 min), after which the supernatant was collected. In the second stage, the sediment was again extracted with corresponding solvent (12 mL) by the same procedure and both supernatants were combined and stored at −20 °C.

The water extracts were then freeze-dried, while the ethanol extracts were evaporated under vacuum (GeneVac HT-4 Series II, Genevac Ltd., Ipswich, UK) and then freeze-dried. The extracts were then resuspended in water (for the water extracts) or DMSO (C_2_H_6_OS, Sigma Aldrich, Steinheim, Germany) (for the ethanol extracts) to obtain concentrated extracts, i.e., 50 mg dry extract/mL (water extracts) and 35 mg dry extract/mL (ethanol extracts).

### 2.3. Yeast Culture Preparation and Treating of Cells with Spirulina Extracts

The effect of *Spirulina* treatment in vivo was evaluated by analyzing the cell response on a proteome level using yeast *Saccharomyces cerevisiae* as a model organism. The yeast *Saccharomyces cerevisiae* was obtained from the Culture Collection of Industrial Microorganisms held by the Biotechnical Faculty of the University of Ljubljana (Slovenia). Yeast was cultivated in YEPD broth (Sigma Aldrich, St. Louis, MO, USA) (220 rpm, 28 °C) until the stationary phase was reached. The cells were then suspended in 50 mM potassium phosphate buffer (PBS, pH 7.8) [31].

Yeast cells were treated with water or ethanol extracts of fermented and non-fermented *Spirulina*, i.e., 3 mg dry water extract/mL or 2.1 mg dry ethanol extract/mL of yeast suspension. Controls were prepared by treating yeast cells using the same volume of each solvent. After treatment, the samples were incubated for 2 h (220 rpm, 28 °C). In this way, four sets of samples were prepared: yeast culture treated with (1) fermented *Spirulina* water extract (SV), (2) non-fermented *Spirulina* water extract (NFV), (3) fermented *Spirulina* ethanol extract (SE) and (4) non-fermented *Spirulina* ethanol extract (NFE), in addition to the water control sample (KV) and ethanol control sample (KE).

### 2.4. Preparation of Yeast Cell Lysates

The treated yeast culture broths were centrifuged (4000 rpm, 3 min). The supernatant was removed and the residual pellet was washed twice with 50 mM PBS and then frozen at −80 °C until extraction. Prior to extraction, the pellet was thawed, and ABC/urea buffer (300 μL) was added. Extraction was performed using three successive freeze-thaw cycles followed by two cycles of homogenization using zirconia/silica beads. The supernatant was then obtained by centrifugation (15,000× *g*, 4 °C, 30 min).

Protein concentration in the extracts was determined according to the method of Bradford [32] with bovine serum albumin (Sigma Aldrich, St. Louis, MO, USA) as the standard. Absorbance (595 nm) was measured using a Safire 2 microplate reader (Tecan, Männedorf, Switzerland) instrument, and the protein concentrations obtained from the standard calibration curve.

### 2.5. Protein Digestion to Peptides for LC-MS

Dithiothreitol (DTT, Sigma Aldrich, St. Louis, MO, USA) was used for reducing protein samples (20 mM, 45 min) and iodoacetamide (IAM, Sigma Aldrich, St. Louis, MO, USA) for alkylation (40 mM, 45 min). Alkylation was performed in the dark and the reaction was terminated using DTT (20 mM, 45 min). Protein digestion in thermoshaker followed (2 h, 37 °C, 750 rpm) with a freshly prepared enzyme mixture of Trypsin/Lys-C (Promega, San Luis Obispo, CA, USA). The enzyme mixture was added to the protein solution in a ratio of 1:25. Thereafter, the lysate was diluted with ABC buffer (50 mM) (Sigma Aldrich, St. Louis, MO, USA) to reach a Urea concentration of 1 M and further digested (overnight, 37 °C, 750 rpm). Formic acid, (FA, Biosolve, Valkenswaard, The Netherlands) was added at a final concentration of 1% to terminate the digestion.

### 2.6. LC-MS Analysis

Peptide separation was performed using an Ultimate 3000 Rapid Separation UHPLC system (Thermo Scientific, Dionex, Amsterdam, The Netherlands). The analytical column was a PepSep C18 (1.9 μm, 120 Å, ID 75 μm × 150 mm). Samples were desalted using an online C18 trapping column. Elution was performed using a linear gradient from 5% to 35% ACN with 0.1 FA in 90 min with a flow rate of 300 nL per minute. The UHPLC system coupled to a Q-Exactive HF Orbitrap mass spectrometer from Thermo Scientific was used for analysis. Data dependent acquisition was as follows: full MS scan from 250 to 1250 *m/z* at a resolution of 120,000. MS/MS scans of the top 15 most intense ions were followed at a resolution of 15,000.

### 2.7. MS Raw Data Analysis

Proteome Discoverer (PD) version 2.2 was used for protein identification and quantification by analyzing the data dependent acquisition spectra. The search engine Sequest was used in the PD software with the SwissProt database (SwissProt TaxID: 4932, Baker’s yeast (*Saccharomyces cerevisiae*)). The following settings were used for the database search: trypsin as the enzyme, a maximum of 2 cleavages missed, 6 was the minimum peptide length, 10 ppm was the precursor mass tolerance, 0.02 Da was the fragment mass tolerance, modifications of methionine oxidation and protein N-terminus acetylation were dynamic, and modification of cysteine carbamidomethylation was static.

The mass spectrometry proteomics data have been deposited to the ProteomeXchange Consortium via the PRIDE (1) partner repository with the dataset identifier PXD027102 (reviewer account details: username: reviewer_pxd027102@ebi.ac.uk, password: NaG26baj).

Default Label Free Quantitation (LFQ) settings in PD were used for protein quantitation. Briefly, peptide precursor intensities were used for peptide abundances, whereas the total peptide amount was used for normalization. For hypothesis testing, we used background-based ANOVA. The calculations of protein ratios were based on pairwise peptide ratios, and the protein differential abundancy is expressed in log2 fold change.

### 2.8. Bioinformatic Analysis of Data

Principal component analysis (PCA) and Volcano plot were prepared in Proteome Discoverer software using proteins (abundance) identified by PD with high confidence (FDR < 5%) from two biological replicates. The UniProtKB database (https://www.uniprot.org/; 15 July 2021) was used to investigate the role of the proteins. Functional annotation analysis was performed using DAVID (https://david.ncifcrf.gov/; 8 July 2021) and QuickGO (https://www.ebi.ac.uk/QuickGO/; 8 July 2021) software and the Kyoto Encyclopedia of Genes and Genomes (KEGG) to integrate the GO categories (Biological Process, Molecular Function, Cellular Component). All identified protein sequences UniProt accession numbers, including identification and quantification data, are provided in the Supplementary Table S1.

## 3. Results and Discussion

### 3.1. Principal Component Analysis (PCA)

**Figure 1:**
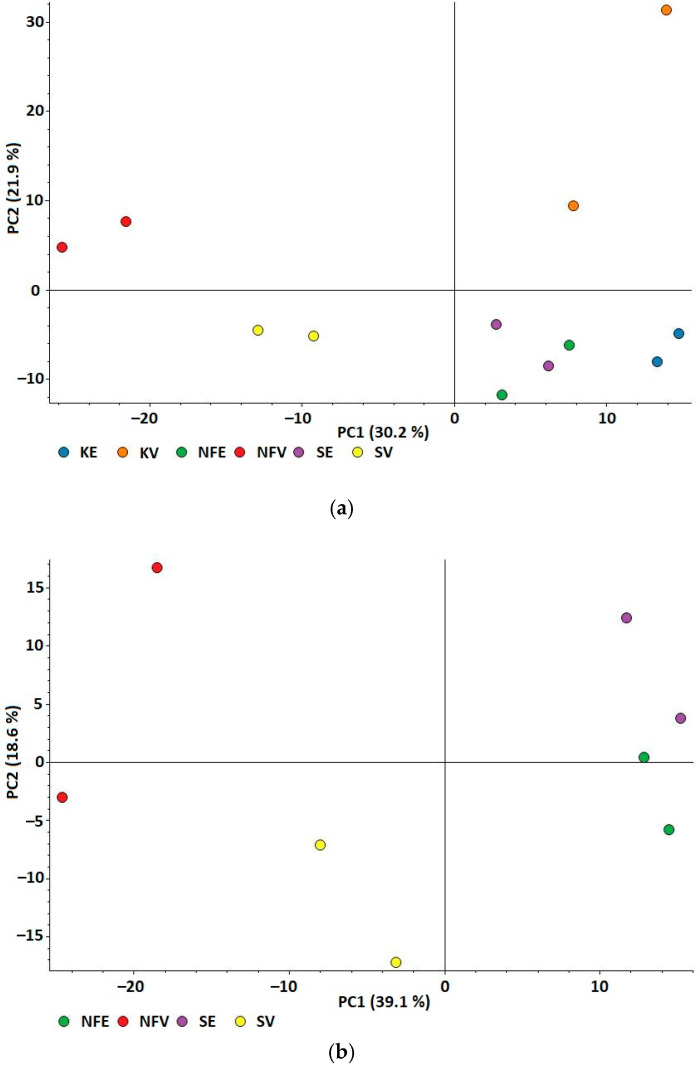
Principal Component Analysis (PCA) of protein abundances. Projection of four sets of samples: yeast treated with fermented *Spirulina* water extract (SV), non-fermented *Spirulina* water extract (NFV), fermented *Spirulina* ethanol extract (SE), and non-fermented *Spirulina* ethanol extract (NFE); and two sets of control samples: water control sample (KV) and ethanol control sample (KE). (**a**) PCA of all samples; (**b**) PCA of samples without control samples.

Principal component analysis (PCA) of the treated samples and controls was used to assess the similarity of proteome profiles (Figure 1) and demonstrate the relationship between yeast samples treated with fermented and non-fermented *Spirulina*, samples treated with water and ethanol extracts and between treated yeast samples and control samples. Two principal components explained the variance of the results. PCA showed the relationship between all samples: component 1 explained 30.2% and component 2 explained 21.9% (Figure 1a) and PCA showed samples without control samples: component 1 explained 39.1% and component 2 explained 18.6% (Figure 1b). The PCA plot shows a clear separation between the *Spirulina* water extract-treated cells and the *Spirulina* ethanol extract-treated cells (including control samples). In addition, a clear separation between the cells treated with *Spirulina* water extracts and water control sample and good separation between the cells treated with ethanol extracts and the ethanol control samples can be observed (Figure 1a). Furthermore, a clear separation between non-fermented *Spirulina*- and fermented *Spirulina* water extract-treated cells can be seen, whereas the separation between non-fermented *Spirulina*- and fermented *Spirulina* ethanol extract-treated cells is less apparent but still distinguishable. A very good separation of the *Spirulina* water extract-treated samples versus the *Spirulina* ethanol extract-treated samples was obtained (Figure 1b) regardless of whether non-fermented or fermented extracts were applied.

PCA analysis of samples indicated the important effect of extraction solvent and lactic acid fermentation on yeast protein profile. For this reason, we made a comparison of the quantitative protein profiles of cells treated with fermented and non-fermented *Spirulina* extracted using water and ethanol.

### 3.2. Quantification and Identification of Differentially Abundant Proteins

**Figure 2:**
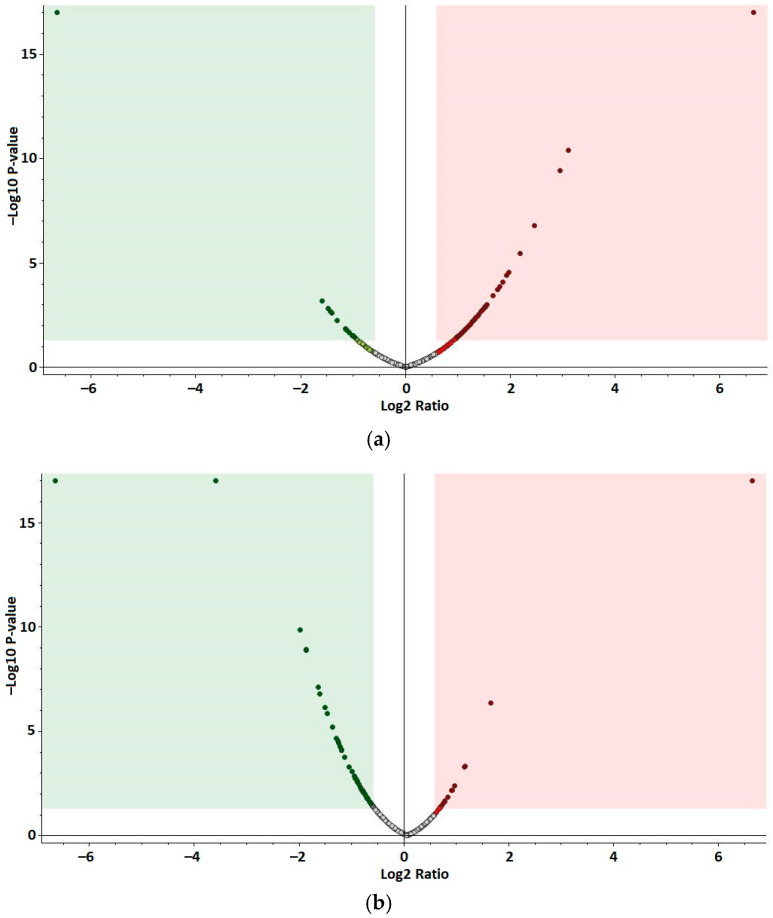
Volcano plots showing differentially abundant proteins in treated yeast samples. The −log10 (*p*-value) is plotted against the log2 fold change. Points above the horizontal line (non-axial) present proteins with significantly different abundances (*p*-value < 0.05). Points to the left of the vertical line (non-axial) present protein log2 fold changes lower than −0.58, and points to the right of the vertical line (non-axial) present protein log2 fold changes greater than 0.58. Significantly downregulated and upregulated proteins *p* value < 0.05) are plotted in the green (left) and red (right) fields, respectively. (**a**) Yeast treated with water extract of fermented *Spirulina* (SV) vs. yeast treated with water extract of non-fermented *Spirulina* (NFV). Fold changes of SV/NFV. (**b**) Yeast treated with ethanol extract of fermented *Spirulina* (SE) vs. yeast treated with the ethanol extract of non-fermented *Spirulina* (NFE). Fold changes of SE/NFE.

The quantitative proteomic analysis identified 641 proteins, of which 91 resulted as differentially abundant (*p*-value ≤ 0.05, log2 fold change ≥ |0.58|)) in SV compared to NFV samples. Among them, 71 proteins were upregulated and 20 proteins were downregulated (Figure 2a). Furthermore, 643 proteins were identified when comparing SE and NFE. Eighty proteins were differentially abundant (*p*-value ≤ 0.05, log2 fold change ≥ |0.58|)), of which 18 were upregulated and 62 were downregulated (Figure 2b). All identified proteins, including differentially abundant proteins, and data referring to PD identification and quantitation are presented in the Supplementary Table S1.

**Figure 3:**
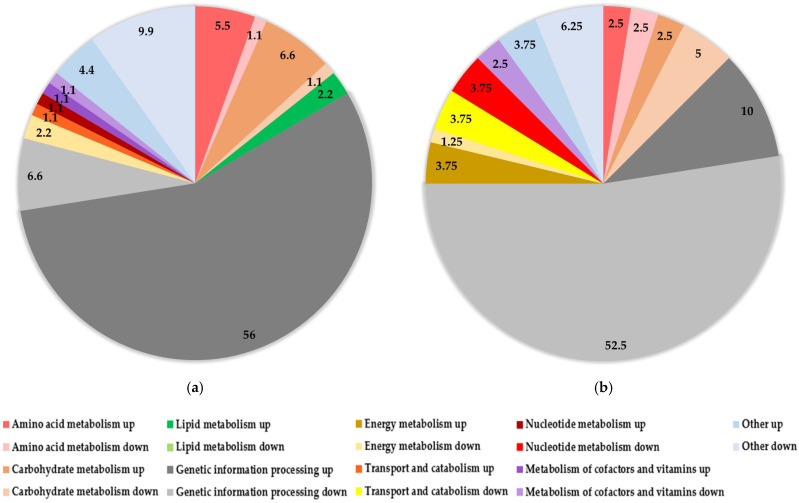
Pie charts showing differentially abundant protein profiles in yeast treated with fermented *Spirulina* extract (S) compared to yeast treated with non-fermented *Spirulina* extract (NF). Results are expressed in percent (%) of significantly upregulated or downregulated proteins involved in different cellular processes. The darker shade of the same color represents the upregulated proteins involved in a specific cell process, and the lighter shade of the same color represents the downregulated proteins involved in the same cell process. (**a**) Yeast treated with water extracts (SV compared to NFV); (**b**) Yeast treated with ethanol extracts (SE compared to NFE).

Further bioinformatic analysis was also performed to obtain more insight into the activity of the differentially abundant proteins. In SV, compared to NFV samples, significantly upregulated proteins (78%) are involved in different cellular processes. Among them, proteins involved in genetic information processing were the most abundant group (56%), followed by those involved in carbohydrate metabolism (6.6%), amino acid metabolism (5.5%), lipid metabolism (2.2%), transport and catabolism (1.1%), nucleotide metabolism (1.1%), and metabolism of cofactors and vitamins (1.1%). Twenty-two percent of significantly expressed proteins show downregulation in SV compared to NFV samples (Figure 3a). Among them are proteins involved in genetic information processing (6.6%), energy metabolism (2.2%), carbohydrate metabolism (1.1%), amino acid metabolism (1.1%), and metabolism of cofactors and vitamins (1.1%).

Conversely, when the proteomic profile of the yeast cells treated with ethanol extracts was analyzed, the opposite situation is observed. The SE samples, when compared to the NFE samples, showed a significant downregulation for the majority (77.5%) of the analyzed proteins involved in different yeast cell processes: genetic information processing (52.5%), carbohydrate metabolism (5.0%), transport and catabolism (3.75%), nucleotide metabolism (3.75%), amino acid metabolism (2.5%), metabolism of cofactors and vitamins (2.5%), and energy metabolism (1.25%). The remaining 22.5% significantly expressed proteins were upregulated in SE samples (Figure 3b) and were associated with genetic information processing (10.0%), energy metabolism (3.75%), carbohydrate metabolism (2.5%), and amino acid metabolism (2.5%).

This turn in cell response when comparing water and ethanol extracts could result from differences in solubility of bioactive compounds from *Spirulina* in both solvents and also from fermentation effect on the bioactive compound release from *Spirulina* and their structural changes affecting their solubility in particular solvent.

*Spirulina* ethanol extracts have been determined to have a higher total phenolic and flavonoid content and higher concentrations of carotenoids and chlorophyll-a compared to water extracts. The compounds responsible for most of the antioxidant activity appear to be polar phenolic compounds, zeaxanthin and myxoxanthophyll-like compounds [16,23,33]. Water extracts, although having lower antioxidant activity, have also been shown to have a beneficial effect on cancer cell growth inhibition, a protective effect against apoptotic cell death due to free radicals, type 2 diabetes managing properties and help improve many other physiological disorders due to their antioxidant activity [3,7,34,35]. They also had higher concentrations of phycocyanin, free amino acids, carbohydrates, and total proteins compared to *Spirulina* ethanol extracts [23].

Studies have shown that an increasing amount of ethanol in the extraction solvent up to 70% also increases antioxidant activity of the *Spirulina* extract, as measured by DPPH (2,2-Diphenyl-1-picrylhydrazyl hydrate) free radical scavenging assay, β-carotene bleaching method, ABTS^•+^ assay or PMRC method [16,22]. This points to the higher solubility of the antioxidative compounds in ethanol and the possibility that ethanol soluble components possess the main antioxidant properties [22,33]. Furthermore, ethanol extracts of different materials (*Spirulina*, green propolis, plants) showed superior antioxidant activity compared to the water extracts [16,22,36,37,38]. In addition, they benefit from ethanol GRAS (Generally Recognized as Safe) status, meaning it can be used as a safe solvent for the food industry [38].

Additionally, in our study, lactic acid bacteria presumably caused modifications of bioactive compounds present in *Spirulina*, which enhanced their ethanol solubility, bioavailability and bioactivity in fermented *Spirulina* extracts. Fermentation has already been shown to improve antioxidant activity by increasing the phenolic compound release from plant-based foods, and it is a suitable method for increasing the content of natural antioxidants. Moreover, during fermentation, the structural breakdown of the cell walls occurs due to the activity of bacterial enzymes. This result is reflected in liberating and/or inducing the synthesis of different bioactive compounds responsible for increasing the total phenols and antioxidant activity after fermentation and facilitating flavonoid extraction [39,40,41]. With protease activity of lactic acid bacteria, phycocyanin, an important antioxidative and anti-inflammatory compound in *Spirulina* can be converted to phycocyanobilin. Other important compounds are bioactive peptides, formed through protein hydrolysis and, together with phycocyanobilin, contribute significantly to fermented *Spirulina* bioactivity [13,42,43]. Structural changes in phytochemicals are another possible mechanism responsible for increasing the bioactivity of plant-based foods after fermentation. The presence of lactic acid bacteria in a fermentation process has been shown to contribute to simple phenolic conversion and depolymerization of high-molecular-weight phenolic compounds [41].

**Figure 4:**
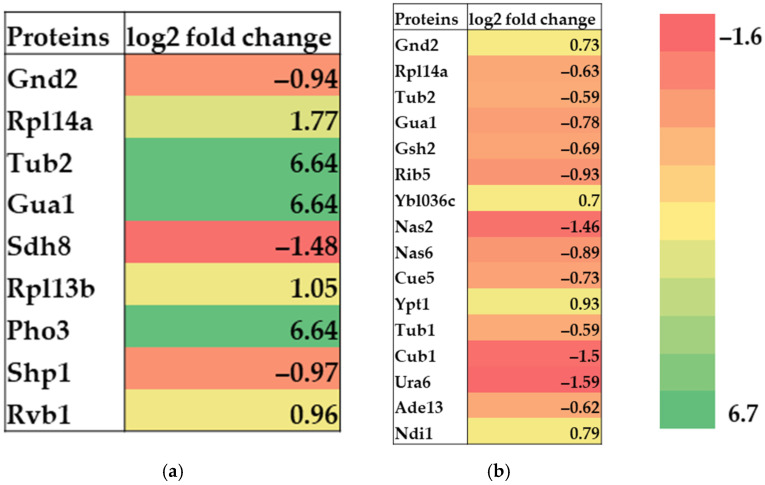
Heat map presenting differentially abundant proteins involved in stress response in yeast treated with fermented *Spirulina* extract (S) compared to yeast treated with non-fermented *Spirulina* extract (NF). Results are expressed as log2 fold change SV/NFV (**a**) and SE/NFE (**b**). Significantly more abundant proteins have log2 fold changes greater than 0.58, and significantly less abundant proteins have log2 fold changes lower than −0.58; *p*-value ≤ 0.05.

To better understand the difference in cell response and to connect it with the antioxidant effect previously determined when comparing the effects of fermented *Spirulina* extracts to non-fermented for particular solvent, we further focused on particular differentially abundant proteins, related to cell stress response and compared their log2 fold changes as an indicator of the degree of abundance (log2 fold changes greater than 0.58 indicate significantly more abundant proteins, and log2 fold changes lower than −0.58 indicate significantly less abundant proteins) (Figure 4). Namely, we used yeast cells in the stationary phase, where, in contrast to the exponential phase (where rapid growth and high biosynthetic activity lead to the dilution of non-repaired lipids or proteins by new and functional ones), the cells are permanently exposed to endogenously produced ROS. This results in accumulation of oxidative damage in the cells [25]. Cell treatment with exogenous antioxidants has already been shown to have effect on endogenous antioxidant defense systems [44].

### 3.3. Stress Response Related Proteins

Six stress response related proteins showed upregulation, and three proteins were downregulated in SV samples compared to NFV samples (Figure 4a). Among them, proteins Pho3, Tub2, and Gua1 showed highly increased levels (6.64). Rpl14a and Rpl13b showed increased values of 1.77 and 1.05, respectively, and Rvb1 had a value of 0.96. Protein Sdh8 had the most decreased level (−1.48), followed by Shp1 (−0.97) and Gnd2 (−0.94). These data show a higher expression of stress response related proteins in yeast treated with fermented *Spirulina* water extract than yeast treated with non-fermented *Spirulina* water extract.

Differentially abundant proteins involved in the stress response of yeast cells treated with *Spirulina* ethanol extracts showed an opposite state. Here, 12 stress response related proteins showed downregulation and 4 proteins were upregulated in the SE samples compared to the NFE samples (Figure 4b). The proteins Ura6 (−1.59), Cub1 (−1.5) and Nas2 (−1.46) showed the highest downregulation, followed by Rib5 (−0.93), Nas6 (−0.89), Gua1 (−0.78), Cue5 (−0.73), Gsh2 (−0.69), Rpl14a (−0.63), Ade13 (−0.62), Tub1 and Tub2 (−0.59). The highest upregulation was noticed in Ypt1 (0.93), followed by Ndi1 (0.79), Gnd2 (0.73) and Ybl036c (0.70). Unlike water extract-treated samples, the data shows the lower expression of stress response related proteins in yeast treated with fermented *Spirulina* ethanol extract than yeast treated with non-fermented *Spirulina* ethanol extract.

The turn in stress response related activity showed as differential expression of stress response related proteins is also shown when comparing four differentially abundant proteins common to both sample groups. Proteins Rpl14a, Tub2 and Gua1, show increased levels in yeast treated with fermented *Spirulina* water extracts, whereas their levels were reduced when fermented *Spirulina* ethanol extracts were applied in comparison with corresponding extracts of non-fermented *Spirulina*. Alternatively, Gnd2 protein was downregulated in yeast treated with water extracts of fermented *Spirulina*, whereas its expression was upregulated in yeast treated with ethanol extracts of fermented *Spirulina* compared to corresponding extract of non-fermented *Spirulina*. The list and names of differentially abundant proteins involved in stress response are presented in Table 1.

A decline in the abundance of Gnd2 protein could result in a higher intracellular oxidation level in SV compared to NFV since this protein is involved indirectly in glutathione metabolism through NADPH production, necessary for maintaining high reduced glutathione (GSH) levels for efficient oxidative stress response [45,46]. Without NADPH, the glutathione oxidized form (GSSG) cannot be reduced to the active form (GSH), causing its levels to drop, which indicates greater cellular oxidative stress [47,48].

Furthermore, upregulation of proteins Rpl14a and Rpl13b might be due to a greater need for degradation of intracellular constituents in SV than NFV samples because these proteins are involved in autophagy. Autophagy is a central part of the integrated stress response mechanism, induced by different stress stimuli: nutrient and energy stress, endoplasmic reticulum stress, pathogen-associated molecular patterns, danger-associated molecular patterns, redox stress, hypoxia, and damage to mitochondria [49,50], which points to a more intense stress response related outcome in the yeast treated with fermented as compared to non-fermented *Spirulina* water extracts.

Additionally, the possibility of DNA damage occurrence and the need for nucleotide base synthesis was indicated in the treated yeast cells since a higher abundance of the DNA damage/DNA repair associated protein Rvb1 was detected in SV compared to NFV samples, as well as upregulation of the Gua1 protein involved in purine biosynthesis. Rvb1 protein has been shown in previous studies to be required for DNA repair and, in higher eukaryotes, it modulates cellular signaling, response to stress and apoptosis and DNA damage [51,52].

An increase in the abundance of Pho3 protein involved in riboflavin metabolism to flavin adenine dinucleotide (FAD) might also indicate a potentially higher oxidation level in SV compared to NFV samples. FAD is a coenzyme with an essential role as an electron acceptor/donor in oxidoreductases such as glutathione peroxidase in *S. cerevisiae*, a FAD-dependent enzyme protecting cells from oxidative stress [53]. Its induction in the treated yeast cells could be related to a higher need for antioxidative action to combat ROS produced in the cell endogenously and cell treatment with *Spirulina* extract.

In contrast to water extract-treated samples, in SE compared to NFE samples, upregulation of the protein involved in glutathione metabolism (Gnd2) indicates a higher NADPH production and consequently a more efficient GSSG reduction to its active form GSH. The result is that GSH predominates in the cells, indicating lower cellular oxidative stress [42,43]. Due to the latter, presumably the need for glutathione biosynthesis also declined, which is seen in our results as downregulation of the Gsh2 protein, relevant for glutathione biosynthesis. It has been suggested in the study of Izawa et al. [54] that *S. cerevisiae* cells adapt to oxidative stress by de novo synthesis of glutathione and glutathione recycling activities, activities which are likely to be reversed when oxidative stress recedes.

Moreover, downregulation of proteins Rpl14a and Cue5 in SE compared to NFE samples might propose a lower occurrence of damage leading to a reduced need for degradation of intracellular components since these proteins are involved in autophagy. This could also be indicated by the downregulation of Nas2, Nas6, and Cub1 proteins associated with the proteasome function, suggesting reduced protein damage and consequently less need for damaged protein degradation in the treated yeast.

Lower abundance of protein Cub 1 which, besides its proteasome function, is also involved in DNA repair, the downregulation of Gua1 and Ade13 proteins associated with purine biosynthesis and Ura6 protein involved in pyrimidine biosynthesis in SE compared to NFE samples might indicate a reduced need for damaged DNA repair and consequently less need for nucleotide base synthesis in the treated yeast cells. Additionally, Rib5, a riboflavin biosynthesis involved protein, was downregulated in SE compared to NFE samples. Riboflavin is an antioxidant nutrient that has antioxidant activity as a component of the glutathione redox cycle or independently, by mechanisms such as converting reduced riboflavin to its oxidized form [55]. A study by Walther and Wendland showed that the synthesis of increased amounts of riboflavin might be connected to oxidative stress exposure in a specific fungus [53]. From this, we might presume that a lower riboflavin biosynthesis is connected to lower oxidative stress exposure.

Downregulation of above mentioned proteins might be a result of a high antioxidant effect of fermented *Spirulina* ethanol extracts. Different studies have shown enhancement of the antioxidant activity of *Spirulina* extracts after fermentation compared to non-fermented *Spirulina* analyzed by scavenging of nitric oxide and the DPPH assay [13,14,56].

## 4. Conclusions

A proteomic approach was used to analyze protein expression alterations in yeast cells treated with fermented or non-fermented *Spirulina* water and ethanol extracts. The results provide a better insight into the chosen extraction solvent effect and the effect of lactic acid fermentation on the antioxidant effect of *Spirulina* previously determined at the cellular level. Proteome analysis showed significant separation between the yeast cells treated with fermented and non-fermented *Spirulina* and between the yeast cells treated with ethanol and water extracts. The results indicated a greater antioxidant efficiency of ethanol than water extracts when comparing fermented to non-fermented *Spirulina* and the essential role of fermentation shown as the lowering of cell stress response related proteins expression. Namely, cells have a reduced need to induce endogenous systems for homeostasis maintenance when they cope with exogenous antioxidants. Further studies, to give an in-depth insight into the *Spirulina* extract effect on the subcellular proteome, are still needed in order to fully understand *Spirulina* bioactive compound mechanism of action. Additionally, our results showed that this kind of approach offers a great potential to study bioactive compounds’ mechanism of action also from other natural sources at a proteome level using a simple eukaryotic cell model.

## Figures and Tables

**Table 1:** Stress response related proteins.

Gene Symbol	UniProt Accession No.	Protein Name	Stress Response Related Activity
GSH2	Q08220	Glutathione synthetase (Gsh2)	Glutathione biosynthesis
GND2	P53319	6-phosphogluconate dehydrogenase, decarboxylating 2 (Gnd2)	Glutathione metabolism
PHO3	P24031	Constitutive acid phosphatase (Pho3)	Thiamine, riboflavin metabolism
RIB5	P38145	Riboflavin synthase (Rib5)	Riboflavin biosynthesis
YBL036C	P38197	Pyridoxal phosphate homeostasis protein (Ybl036c)	Homeostatic regulation of the active form of vitamin B6
SDH8	P38345	Succinate dehydrogenase assembly factor 4, Mitochondrial (Sdh8)	Response to reactive oxygen species
RPL13B	P40212	60S ribosomal protein L13-B (Rpl13b)	Autophagy
RPL14A	P36105	60S ribosomal protein L14-A (Rpl14a)	Autophagy
CUE5	Q08412	Ubiquitin-binding protein CUE5 (Cue5)	Autophagy
YPT1	P01123	GTP-binding protein YPT1 (Ypt1)	Autophagy
SHP1	P34223	UBX domain-containing protein 1 (Shp1)	Autophagosome assembly, proteasome-mediated ubiquitin-dependent protein catabolic process, piecemeal microautophagy of the nucleus
TUB2	P02557	Tubulin beta chain (Tub2)	Phagosome function
TUB1	P09733	Tubulin alpha-1 chain (Tub1)	Phagosome function
RVB1	Q03940	RuvB-like protein 1 (Rvb1)	DNA damage, DNA repair
CUB1	Q08977	Cu^(2+)^ suppressing and bleomycin sensitive protein 1 (Cub1)	DNA repair and/or proteasome function
NAS2	P40555	Probable 26S proteasome regulatory subunit p27 (Nas2)	Proteasome regulatory complex assembly
NAS6	P50086	Probable 26S proteasome regulatory subunit p28 (Nas6)	Proteasome regulatory complex assembly
GUA1	P38625	GMP synthase (glutamine-hydrolyzing) (Gua1)	Purine biosynthesis
ADE13	Q05911	Adenylosuccinate lyase (Ade13)	Purine biosynthesis, AMP, IMP biosynthesis
URA6	P15700	Uridylate kinase (Ura6)	Pyrimidine biosynthesis
NDI1	P32340	Rotenone-insensitive NADH-ubiquinone oxidoreductase, mitochondrial (Ndi1)	Positive regulation of the apoptotic process

## Data Availability

The mass spectrometry proteomics data have been deposited to the ProteomeXchange Consortium via the PRIDE (1) partner repository with the dataset identifier PXD027102 (reviewer account details: username: reviewer_pxd027102@ebi.ac.uk, password: NaG26baj). Other data is contained within the article and supplementary.

## Supplementary Materials

The following are available online at https://www.mdpi.com/2076-3921/10/9/1366/s1, Table S1: Proteome Discoverer protein identification and quantification data.
